# Personal News Items

**Published:** 1915-06

**Authors:** 


### Personal News Items.

Dr. L. M. Davis has been appointed city health officer of Robstown.

Dr. M. B. Grace of Seguin made a business trip to New Mexico the latter part of April.

Dr. J. W. McComb is in Austin, as Representative, during the called session of the Legislature.

Dr. D. A. Simmons has returned to his home in Sherman after spending the winter in California.

Dr. W. G. Covington of North Fort Worth has been appointed to the position of Niles City physician.

Dr. J. C. Phillips of Port Arthur will spend the next six weeks in New York doing post-graduate work.

Dr. W. M. Copeland of Loraine left for Chicago, where he will take a post-graduate course in surgery.

Dr. S. W. Lytal has returned to his home in Quinlan after a pleasant visit in Alabama and Mississippi.

Dr. James Greenwood of Houston was called to Seguin recently because of the serious illness of his father.

Dr. J. W. Johnson, formerly of Chillicothe, has moved to Arlington to take charge of the home for aged Masons.

Dr. T. R. Sealey of Santa Anna, Texas, who has been spending some time in California, is expected home soon.

Dr. W. B. Georgia of Waco is confined to the Providence Sanitarium, where he underwent a serious operation.

Dr. and Mrs. Jesse Robards and children have returned to their home in Clarendon after spending the winter in Chicago.

Dr. W. F. Shepherd of Tatum had the misfortune to get his right arm broken in two places while cranking his automobile.

Dr. Paul Sheppard of Terrell, Texas, has returned from Tulane University, New Orleans, where he has been doing some special work.

Dr. J. H. Loving has returned to his home in Wellington after a sojourn of several months in New Orleans, taking post-graduate work.

Dr. J. E. Wilkins of Greenville was one of the Texas delegates to the Southern Sociological Congress which convened in Houston May 8-11.

Dr. C. F. Johnson of Seymour has gone to St. Paul, Minn., where he will take an exhaustive surgical course with Drs. Mayo of that place.

Dr. J. W. Harwell, widely known cancer specialist of Rockport, who suffered a stroke of paralysis, is rapidly recovering under the skillful care of Dr. Harlan Trask.

Dr. I. L. McGlasson of Waco was one of the Texas delegates appointed by Governor Ferguson to the Southern Sociological Congress which was held in Houston, May 8-11.

Dr. and Mrs. I. F. Orton of Galveston have returned from an extensive trip through the West, visiting San Francisco, Los Angeles, Denver, Salt Lake and other points of interest.

The many friends of Dr. Charles C. Foster are pleased to learn that he has returned to his home in Georgetown after several months' treatment under a specialist in Galveston, much improved in health.

In anticipation of Dallas Clean-up Day, Dr. Manton M. Carrick of Dallas will deliver his address on "The City's Greatest Asset, Health," under the auspices of the People's Central Forum, at an early date.

Dr. W. L. Gaines of Seymour, Texas, who has been doing some post-graduate work in Tulane University, New Orleans, for the past three months, was called home because of the serious illness of his little son.

Dr. Catherine Compton of Beeville, Texas, was called by telegram to Worthington, Ind., because of the sudden death of her mother. Her many friends in Texas deeply sympathize with her in her bereavement.

Dr. C. L. King of Whitesboro has been appointed local and consulting surgeon for the Missouri, Kansas & Texas Railway. He has been local surgeon for the Texas & Pacific Railway for the past twenty-two years.

Dr. C. H. Sanders, formerly of Calvert and Waco, Texas, who was sent to Germany by the American Red Cross in charge of the hospital unit, has returned to the United States after a six months' tour on the duty there.

Dr. and Mrs. Wiley J. Jinkins have returned to Galveston from their wedding trip to Hot Springs, Ark., New Orleans, Little Rock, Ark., and other points, and will be with Dr. and Mrs. H. O. Sappington until the completion of their new home.

---

The following have become members of the TEXAS MEDICAL JOURNAL family and we bid them a most cordial welcome:

Dr. M. R. Sharp, Granger, Texas; Dr. J. N. Nichols, Bangs; Dr. M. Smith, Oklahoma City, Okla.; Dr. A. S. Brown, Abilene; Dr. R. A. Harris, Winnsboro; Dr. C. O. Terrell, Ranger; Dr. R. B. Talbot, Fort Worth; Dr. J. L. Allred, Winters; Dr. R. D. Chunn, Singleville; Dr. J. W. Eads, Barksdale; Dr. M. H. Herndon, Lindon; Dr. Chas. McNally, Weatherford; Dr. J. D. Mickie, Childress; Dr. J. B. Shannon, Fort Worth; Dr. Paul Lipps, Fort Worth; Dr. R. L. Howell, Snyder; Dr. C. R. Gowen, Sterling City; Dr. J. F. Clark, Iowa Park; Dr. A. J. Evans, Caddo; Dr. W. R.

Johnson, Snyder; Dr. J. M. Vanness Prairie Lea; Dr. J. G. Webb, Fort Worth; Dr. Oscar Dowling, State Health Officer, New Orleans, La.; Dr. A. J. Caldwell, Corpus Christi; Dr. J. S. Wilkins, Paducah; Dr. J. B. Dunn, Hubbard; Dr. V. E. Robbins, Quitman; Dr. Chas. F. Rice, Gainesville; Dr. H. V. Reeves, Crandall; Dr. G. P. Pipkin, Dallas; Dr. W. C. Swain, Dallas; Dr. J. H. Black, Dallas; Dr. Ed Graves, Gatesville; Dr. R. C. Youngblood, Falls City, Texas.

